# Composition of the Gut Microbiome Influences Production of Sulforaphane-Nitrile and Iberin-Nitrile from Glucosinolates in Broccoli Sprouts

**DOI:** 10.3390/nu13093013

**Published:** 2021-08-28

**Authors:** John A. Bouranis, Laura M. Beaver, Jaewoo Choi, Carmen P. Wong, Duo Jiang, Thomas J. Sharpton, Jan F. Stevens, Emily Ho

**Affiliations:** 1 School of Biological and Population Health Sciences, Nutrition Program, Oregon State University, 101 Milam Hall, Corvallis, OR 97331, USA; bouranij@oregonstate.edu (J.A.B.); laura.beaver@oregonstate.edu (L.M.B.); carmen.wong@oregonstate.edu (C.P.W.); 2 Linus Pauling Institute, Oregon State University, 307 Linus Pauling Science Center, Corvallis, OR 97331, USA; jaewoo.choi@oregonstate.edu (J.C.); fred.stevens@oregonstate.edu (J.F.S.); 3 Department of Statistics, Oregon State University, 239 Weniger Hall, Corvallis, OR 97331, USA; Duo.jiang@oregonstate.edu (D.J.); thomas.sharpton@oregonstate.edu (T.J.S.); 4 Department of Microbiology, Oregon State University, 226 Nash Hall, Corvallis, OR 97331, USA; 5 Department of Pharmaceutical Sciences, Oregon State University, 1601 SW Jefferson Way, Corvallis, OR 97331, USA

**Keywords:** bacteria, glucosinolate, iberin, iberin-nitrile, isothiocyanate, microbiome, sulforaphane, sulforaphane-nitrile

## Abstract

Isothiocyanates, such as sulforaphane and iberin, derived from glucosinolates (GLS) in cruciferous vegetables, are known to prevent and suppress cancer development. GLS can also be converted by bacteria to biologically inert nitriles, such as sulforaphane-nitrile (SFN-NIT) and iberin-nitrile (IBN-NIT), but the role of the gut microbiome in this process is relatively undescribed and SFN-NIT excretion in humans is unknown. An ex vivo fecal incubation model with in vitro digested broccoli sprouts and 16S sequencing was utilized to explore the role of the gut microbiome in SFN- and IBN-NIT production. SFN-NIT excretion was measured among human subjects following broccoli sprout consumption. The fecal culture model showed high inter-individual variability in nitrile production and identified two sub-populations of microbial communities among the fecal cultures, which coincided with a differing abundance of nitriles. The Clostridiaceae family was associated with high levels, while individuals with a low abundance of nitriles were more enriched with taxa from the Enterobacteriaceae family. High levels of inter-individual variation in urine SFN-NIT levels were also observed, with peak excretion of SFN-NIT at 24 h post broccoli sprout consumption. These results suggest that nitrile production from broccoli, as opposed to isothiocyanates, could be influenced by gut microbiome composition, potentially lowering efficacy of cruciferous vegetable interventions.

## 1. Introduction

Cruciferous vegetables, including broccoli, brussels sprouts, collard greens, and arugula, ubiquitously contain a class of compounds known as glucosinolates (GLS) [1]. Following damage to the cell wall of the vegetable, either the plant enzyme myrosinase converts GLS to bioactive isothiocyanates (ITC) or the epithiospecifier protein can facilitate conversion to nitriles (NIT) [1,2,3,4]. Broccoli sprouts are known to be rich in the aliphatic GLS glucoraphanin and also contain glucoiberin, which yield the ITCs sulforaphane (SFN) and iberin (IBN), respectively, as well as the nitriles sulforaphane-nitrile (SFN-NIT) and iberin-nitrile (IBN-NIT) [2]. Cell culture and animal models have shown that aliphatic ITCs, such as SFN and IBN, have potent anti-cancer properties and can suppress and prevent the formation of cancer [5,6,7,8,9,10,11,12,13,14,15,16]. For example, SFN can inhibit cancer cell growth, promote cancer cell apoptosis, decrease inflammation and angiogenesis, and inhibit dysregulated enzymes that regulate epigenetics [8,10,11,14,16,17,18,19]. NITs, on the other hand, are produced from the same GLS, but are widely considered biologically inert, although, this is becoming controversial [5,20,21,22,23]. SFN-NIT excretion in humans following cruciferous vegetable consumption has not been previously described and, thus, the extent that it is present and biologically relevant is unknown.

At the population level, epidemiological studies have shown an inverse relationship between the incidence of lung, breast, prostate, colon, and bladder cancer and cruciferous vegetable consumption, offering an appealing, cost-effective, non-pharmacological approach to cancer prevention through dietary intervention [24,25,26,27]. In contrast to these findings, human clinical trials examining the efficacy of cruciferous vegetables interventions on cancer prevention targets showed high levels of inter-individual variation in both the absorption and excretion of ITCs, however, the source of this variation is unknown [28]. Early work has focused on glutathione-S-transferase polymorphisms as a major factor modulating the effects of ITC metabolism and variable effects on cancer, but studies are inconsistent and equivocal [29,30,31,32,33,34,35,36,37,38,39,40,41]. Our own group showed that *Nrf2* KO mice, which lack the ability to induce *glutathione-S-transferases*, do not manifest differences in SFN metabolite production [42]. As an alternative, it was proposed that differences in individual gut microbiomes may contribute to the observed variation in ITC levels, potentially through the production of biologically inert NITs from GLS by gut microbes. This is significant as an increase in NIT production from GLS reduces the amount of ITCs produced and, thus, may lower the efficacy of cruciferous vegetables interventions.

Members of the human commensal gut microbiota have been shown to hydrolyze GLS to both ITCs and NITs in the absence of myrosinase, such as in cooked vegetables [12,43,44,45]. Preliminary studies conducted in humans fed glucoraphanin showed that variations in SFN excretion coincide with variations in gut microbiome composition [46,47]. In addition to this evidence, in vitro studies demonstrated that different microbes preferentially hydrolyze GLS to either ITCs or NITs [48,49,50,51]. Additionally, consumption of cruciferous vegetables can significantly alter the composition of the gut microbiome, highlighting the potential diet-microbiome effects [46,47,52,53,54]. Furthermore, the gut microbiome has been implicated in tumorigenesis, thus, understanding shifts to gut microbial communities in response to cruciferous vegetable consumption may give further insights into the anti-cancer properties of cruciferous vegetables [55,56]. It is hypothesized that the gut microbiome is the underlying driver of the variation observed in many human clinical trials, however, evidence on these complex and dynamic systems is lacking. More specifically, work on the role of the gut microbiome in GLS metabolism is limited primarily to in vitro studies conducted using GLS-isolates in monocultures of culturable organisms. These studies fail to capture both the impacts of a whole food matrix on the microbial metabolism of GLS, and the effects of broccoli consumption on microbiome composition.

To further investigate the role of the gut microbiome in the production of NITs from GLS, we utilized an ex vivo fecal incubation model using individual human stool samples and broccoli sprouts that underwent in vitro digestion. To verify SFN-NIT was detectable among human subjects, SFN-NIT excretion following broccoli sprout consumption was measured. Despite a growing interest in how the microbiota affects dietary chemoprevention agents, few studies have closely examined the interaction among the gut microbiota and GLS in complex and dynamic systems. The results of this study will assist in understanding determinants of individual response to cruciferous vegetable metabolism and responses to dietary interventions.

## 2. Materials and Methods

### 2.1. Ex Vivo Fecal Incubation Model: In Vitro Broccoli Sprouts Digestion and Ex Vivo Human Fecal Culture

Broccoli sprouts were chosen for this study as they are a rich source of glucoraphanin and contain glucoiberin, as well as other microbiome-modulatory compounds, such as fiber and flavanols. Broccoli sprouts were grown from broccoli seeds (Sprout House, Lake Katrine, NY, USA) as published in [57,58,59,60]. The sprouts were harvested on day four, crushed with a mortar and pestle to mimic chewing, and in vitro digested using an oral, gastric, and intestinal phase as described elsewhere [61,62,63,64,65]. Briefly, samples were crushed with a mortar and pestle before salivary amylase was added to simulate oral phase of digestion. Samples were then acidified to a pH of 2.5 with hydrochloric acid and pepsin was added to simulate the gastric phase of digestion. Lastly, sodium hydroxide was added to neutralize the samples to a pH of 7 and bile salts, pancreatin, and mucin were added for the intestinal phase of digestion. For fecal bacterial cultivation, a 20% fecal slurry was made from fecal stocks that consisted of fecal material from 10 healthy volunteers (six female and four male, age 17–51, Lee Biosolutions) and sterile PBS (0.1 M pH 7). A total of 500 µL of fecal slurry was mixed with 10 mL of brain heart infusion (BHI) broth, with hemin and vitamin K, per the manufacturer’s recommendation, and either 500 µL of filter-sterilized in vitro digested broccoli sprouts (Broc) or a negative control (NC) in vitro digestion. The NC contained reverse osmosis water, equivalent in volume to the water content of broccoli sprouts, and underwent the same in vitro digestion procedure as described above with the same enzymes, chemicals, and equipment. Broc digest was scaled to be equivalent in concentration to a human consuming a ½ cup of broccoli sprouts. Cultures were incubated at 37 °C for 24 h in anaerobic conditions [66]. The fecal culture medium was then vortexed, sampled, centrifuged (13,000× *g* for 10 min) and the supernatants were frozen in liquid nitrogen. Fecal samples, post-incubation, were also collected and stored at −80 °C for microbiome analysis.

### 2.2. Human Urine Samples

The human feeding trial was conducted at Oregon State University. Study protocols were approved by the Institutional Review Board at OSU (OSU IRB #4995). Human urine samples were from a published human feeding trial [14]. Briefly, ten healthy adults were recruited in Corvallis, Oregon. Exclusion criteria included smoking, BMI < 18.5 and > 30 kg/m^2^, vegetarianism, and the use of drugs that alter lipid metabolism. Fasting subjects consumed one serving of fresh broccoli sprouts, containing 200 µmol of SFN equivalents, along with a breakfast of bagel, cream cheese, orange juice, milk, or coffee. The same breakfast was provided at 24 and 48 h without sprouts. During the duration of the study, and seven days prior, participants were asked to discontinue consumption of all cruciferous vegetables and phytochemical supplements. After breakfast, no other food was provided, and participants self-selected food. Urine samples were obtained before broccoli sprout consumption (0 h) and complete urine was collected between 0–3, 3–6, 6–12, 12–24, and 24–48 h post-consumption. While in the subjects’ possession, urine was refrigerated or kept on ice in opaque jugs containing granulated boric acid (~20 mg/mL) to stabilize SFN metabolites. Upon receipt, the urine was acidified with TFA to a final concentration of 10% *v*/*v* and saved in urine storage tubes at −80 °C.

### 2.3. Microbial Sequencing

DNA was isolated from fecal stocks (pre-incubation) and fecal cultures (post-incubation) using a QIAamp PowerFecal DNA kit (Qiagen, Hilden, Germany) per the manufacturer’s protocol. The fecal DNA concentration was measured using a Qubit dsDNA HS assay kit (Invitrogen, Waltham, MA, USA). PCR was used to amplify the 16S rRNA gene at the V4V5 region, then sequenced using an Illumina MiSeq to produce a sequence library using the Earth Microbiome Project protocol [67]. This approach yielded 300 bp, paired end amplicon sequences at a target sequencing depth of 50,000 reads per sample. The 16S amplicon sequencing was done at the Center for Quantitative Life Sciences core facilities (Oregon State University, Corvallis, OR, USA) using established methods [68]. Data preprocessing and identification of amplicon sequence variations (ASVs) was conducted using the DADA2 pipeline, as implemented in R (v3.5) [69]. Briefly, the reads were first trimmed for read quality and filtered for two expected errors, followed by a margining of paired reads and a removal of chimeric ASVs. The taxonomy was assigned using the Silva database V132 with the naïve Bayesian classifier built into DADA2 [69,70].

### 2.4. Isothiocyanate Quantification

Metabolites from fecal culture medium were extracted (100 μL culture/100 μL ice cold 80:20 methanol:water), mixed vigorously, and clarified by centrifugation (13,000× *g* for 10 min). The supernatants were further diluted 1:10 with ice cold 80:20 methanol:water (*v*/*v*) and transferred to mass spectrometry (MS) vials. SFN-NIT and IBN-NIT were detected using LC-MS/MS in positive ion mode, as previously described [71,72]. Briefly, HPLC was performed on a Shimadzu Nexera system (Shimadzu, Kyoto, Japan) with a phenyl-3 stationary phase column (Inertsil Phenyl-3, 5 µm, 4.6 × 150 mm; GL Sciences, Tokyo, Japan) coupled to a quadrupole time-of-flight MS (AB SCIEX TripleTOF 5600). The samples were randomized, auto-calibration was performed every two samples, and a quality control sample, composed of a pooled aliquot from each sample, was analyzed every 10 samples. MS/MS information was obtained for all samples using information dependent acquisition (IDA), while sequential window acquisition of all theoretical spectra (SWATH) was performed only on quality control samples. The data were analyzed using PeakView with XIC Manager 1.2.0 (ABSciex). SFN-NIT and IBN-NIT identities were assigned by matching the accurate mass (error < 5 ppm), retention time (error < 5%), isotope distribution (error < 20%), and MS/MS fragmentation pattern against commercially available standards for SFN-NIT and IBN-NIT (LKT Labs and Millapore). The peak list was exported to MultiQuant 3.0.2 (SCIEX) to integrate chromatograms and obtain peak areas.

To quantify the amount of SFN-NIT and SFN-N-acetyl cysteine (SFN-NAC) in the urine from the human subjects, we utilized a targeted liquid chromatography and mass spectrometry analysis. The urine was acidified to a final acid concentration of 10% (*v*/*v*) to allow the proteins to precipitate. After 5 min, samples were centrifuged at 16,000× *g* for 5 min to pellet proteins. The recovered supernatant was directly injected for analysis on a SCIEX-4000 QTRAP LC-MS/MS instrument using a Phenomenex Kinetex 2.6µ PFP, 100A pentafluorophenyl (PFP) column. The resulting peaks were quantified using a standard curve of SFN-NIT and SFN-NAC (LKT Labs). The total urine volume was used to calculate the µmol of SFN-NAC and SFN-NIT excreted.

### 2.5. Data Management and Quantification of ASVs

All statistical analysis was conducted in R version 4.1.0, unless otherwise noted. The Benjamini-Hochberg procedure was used for multiple testing correction and an adjusted *p*-value of 0.05 was used as the significance threshold [73]. Using phyloseq, the ASVs were first agglomerated at the genus level, reducing the number of ASVs from 3557 to 935 due to a high number of unannotated ASVs [74]. To remove noise from the dataset, highly rare genera not seen more than three times in at least 20% of the samples and with a relative abundance less than 0.001%, were filtered out, resulting in a final dataset of 72 ASVs. Rarefaction curves, using the vegan package, were built on agglomerated and filtered data to ensure all samples were sufficiently sequenced (Figure S1) [75].

### 2.6. Diversity Analysis and Visualization

Phyloseq and ggplot2 were used to visualize and calculate alpha-diversity metrics using the unfiltered, un-agglomerated data [74,76]. Differences in alpha-diversity were assessed using non-parametric tests and generalized linear mixed models to account for repeated measures, when necessary. Dunn’s test was used for post-hoc testing and Benjamini-Hochberg procedure was used for multiple testing correction [73,77,78]. Specific models used are described in the results.

### 2.7. Beta-Diversity Analysis 

Beta-diversity of agglomerated and filtered data was analyzed using principal coordinate analysis (PCoA) and the ordinations were calculated by phyloseq based on Bray-Curtis distance [74]. Permutation analysis of variance (PERMANOVA) was conducted using the adonis function from the vegan package [75]. To identify sub-communities in our samples, k-means clustering using the Hartigan-Wong algorithm was conducted on the PCoA using 25 random starts [79]. The optimal number of clusters was selected using the elbow method.

### 2.8. Discriminant Analysis

To identify bacterial taxa associated with the clusters detected by k-means, we utilized a linear discriminant analysis effect size (LEfSe), as implemented by the browser-based Galaxy Module [80]. An alpha value of 0.05 was used for the LEfSe Kruskal-Wallis and Wilcoxon tests with a logarithmic LDA score threshold of two. k-means cluster was used as the class, with treatment as the sub-class. In addition to LEfSe, we utilized a negative binomial general linear mixed-effect model, as implemented by the package lme4, to identify taxa that were differentially abundant between the two clusters [81]. All samples were rarefied to an even depth prior to fitting the model. The abundance of each ASV was used as the response variable, with the interaction of treatment and k-mean cluster used as the predictor variables. One model was built for each ASV with relative abundance as the response variable and the interaction of treatment and k-mean cluster as the predictor variables. The Benjamini-Hochberg procedure was used to account for multiple tests [73].

### 2.9. Correlation Analysis

To identify correlations between SFN-NIT, IBN-NIT, and the abundance of each taxa measured, we utilized Spearman’s correlation. First, the taxa were agglomerated to the family-level using phyloseq. Then, pairwise correlations were conducted between each family and the abundance of SFN-NIT and IBN-NIT in each sample [74].

### 2.10. Code Availability

R code is available at github: https://github.com/bouranij/Nutrients_GLS_Nitriles (accessed on 20 August 2021).

## 3. Results

### 3.1. Characteristics of the Pre-Incubation Fecal Slurries

**Figure 1:**
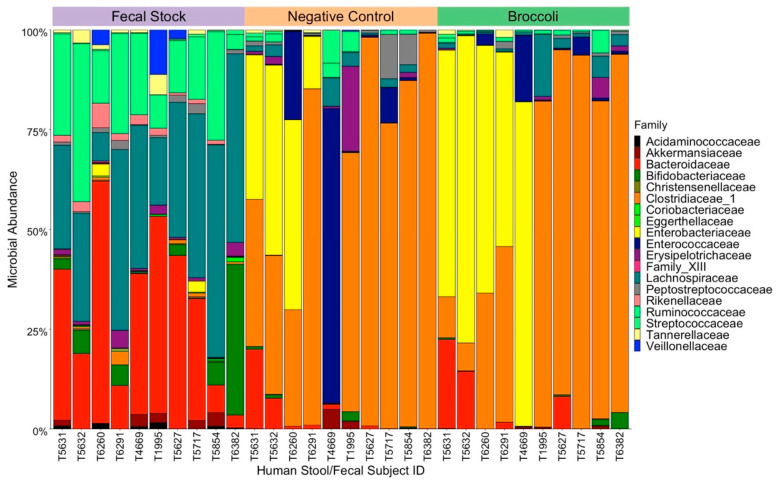
Relative abundance of each family within the pre- and post-incubated fecal cultures (*n* = 10). Each color corresponds with a different family and the size of each bar corresponds to relative abundance of that family within each sample. Bars at the top represent treatment groups, pre-incubation fecal stocks (fecal stocks), post-incubation with in vitro digested broccoli (Broc), and post-incubation negative control digest (NC).

To evaluate the overall microbial composition of each donor fecal sample, we calculated the alpha-diversity using the observed species richness, Shannon, and Simpson measures. Shannon and Simpson measures of alpha-diversity showed relatively low levels of variation, with Shannon diversity ranging from 5.281 to 6.29 and Simpson diversity ranging from 0.991 to 0.997 (Table S1). Conversely, observed richness showed the greatest level of variation between subjects, ranging from 364 to 696 observed taxa (Table S1). The most abundant families in the fecal stocks were Bacteroidaceae, Lachnospiraceae, and Ruminococcaceae, which cumulatively composed over half the taxa present in each sample (Figure 1). There were no significant differences by age nor biological sex for all measures of alpha-diversity (Table S2). To examine compositional differences between the samples, we examined beta-diversity using a principal coordinate analysis (PCoA) (data not shown). Neither age (binned in 10-year intervals) nor biological sex were found to be significant as determined by a permutation analysis of variance (PERMANOVA) test, indicating that these factors were not driving differences in microbial composition between fecal stocks.

### 3.2. Incubation with Broccoli Sprout Digest Does Not Alter Gut Microbial Composition

To investigate the impact of broccoli sprouts on the gut microbiome, the microbial composition of fecal culture samples following incubation with Broc or NC digest was analyzed (Figure 1). For all measures of alpha-diversity, there was a significant difference between the pre-incubation fecal stocks and both the Broc and NC samples. No significant differences in alpha-diversity were found between the Broc and NC treatments, indicating that incubation with broccoli did not lead to a reduction in the diversity of microbes present in our samples, relative to a control, a hallmark of poor gut health (Table S3). It is worth noting that sample T4669 changed drastically in composition between NC and Broc treatments.

**Figure 2:**
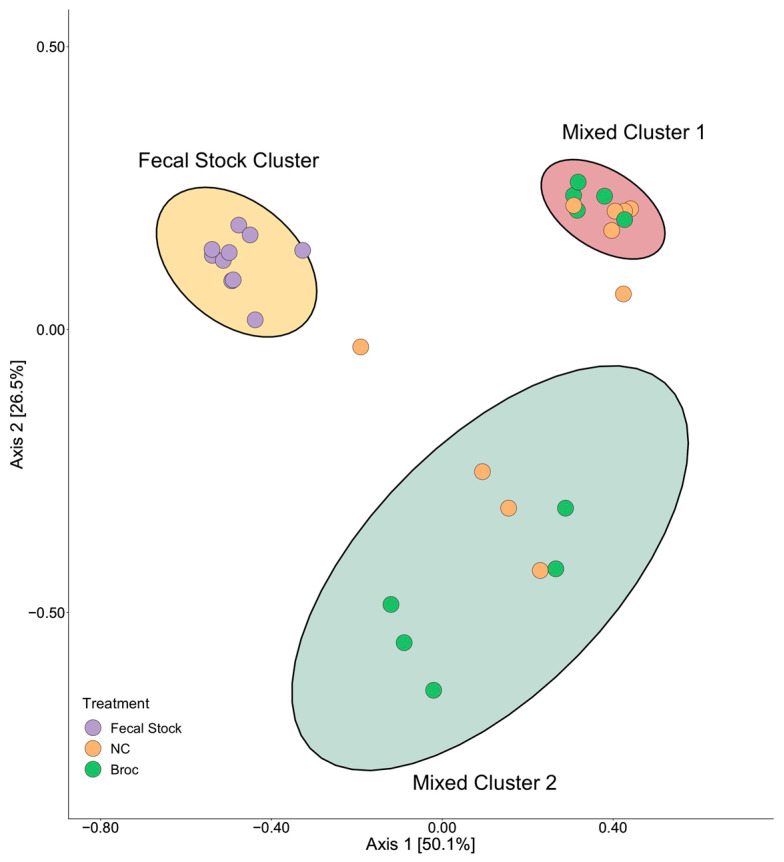
Principal coordinate analysis (PCoA) showing beta-diversity of samples. Each point represents one sample, with the color of the point indicating if it is a pre-incubation fecal stock or post-incubation fecal culture treated with in vitro digested broccoli (Broc), or a negative control (NC) in vitro digest. Clusters, indicated by colored ellipses, were verified by k-means clustering and the number of clusters selected by the elbow method.

To evaluate how incubation with broccoli sprouts impacted microbial diversity and abundance between samples, beta-diversity between fecal stocks and post-incubation samples was analyzed. The resulting PCoA contained three clusters of samples (Figure 2): one cluster contained all the pre-incubation samples, and two additional clusters, each of which contained a mix of both Broc and NC samples. To verify the observed clusters, k-means clustering on the PCoA with 25 random starts and using between 2 and 10 clusters was conducted. The elbow method on the within-clusters sum of squares verified the presence of three clusters within our bacterial samples. As expected, one of these clusters was composed entirely of pre-incubation fecal stock samples, except for a single outlying NC sample. Consistent with the PCoA findings, the other two clusters were composed of a mix of both NC and Broc samples (Figure 2).

To further investigate the two observed mixed clusters, the pre-incubation fecal stock samples were removed and a new PCoA was formed (Figure S2). In the post-incubation PCoA, the two clusters previously observed persisted and were once again verified by k-means clustering. Treatment was found to be non-significant (*p* = 0.55) by PERMANOVA, verifying our previous observation that the clustering observed was not driven by the incubation of broccoli with the fecal stocks. Conversely, sample donor was found to be significant (*p* = 0.001) by PERMANOVA. Cumulatively, these findings indicated that, while anaerobic incubation significantly altered the composition of the microbiome, broccoli sprouts did not significantly alter the microbiome relative to the negative control digest. Additionally, we identified two sub-populations within our samples, regardless of exposure to broccoli sprouts.

### 3.3. Gut Microbiome Composition Influences Nitrile Production

**Figure 3:**
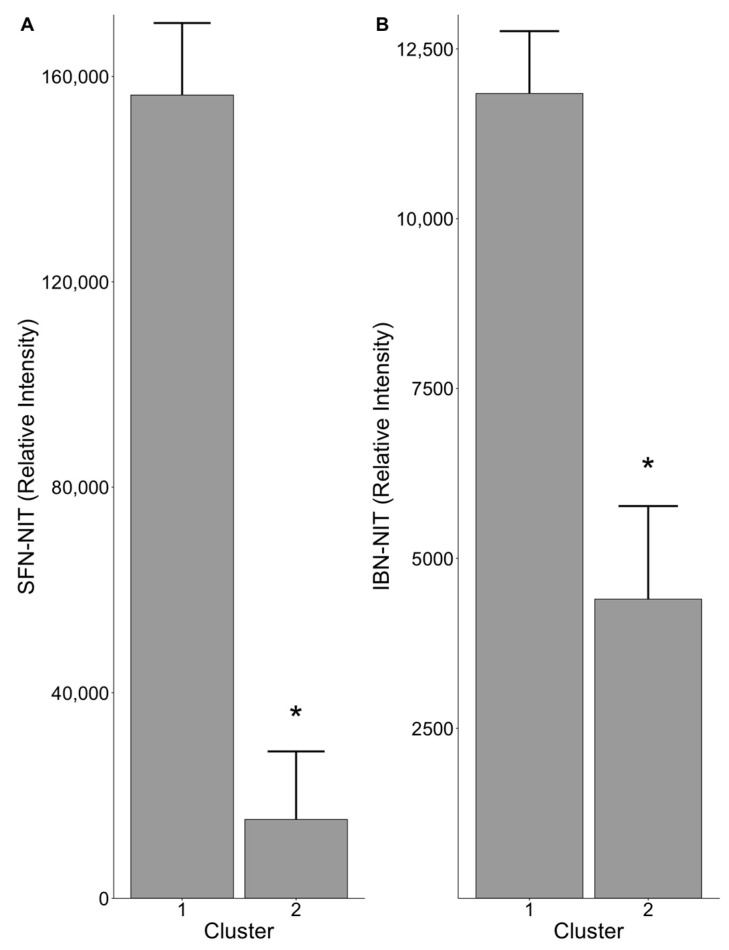
(**A**) SFN-NIT and (**B**) IBN-NIT levels detected in fecal cultures (*n* = 5/cluster) following anaerobic incubation with broccoli sprouts for 24 h. Cluster corresponds to the microbial communities detected through PCoA analysis (Figure 2). Error bars show SEM. Asterisks denote statistically significant differences (*p* < 0.05).

Using mass spectrometry, we measured the abundance of SFN-NIT and IBN-NIT in our samples. The relative abundance of SFN-NIT in the fecal culture medium, measured by integrating their chromatographic peak areas, was significantly different (*p* = 0.009) between samples belonging to clusters 1 and 2, with the relative intensity being 156,400 and 15,336 for each cluster, respectively. It was also significantly different (*p* = 0.009) for IBN-NIT, with the relative intensity being 11,844 and 4399 for cluster 1 and 2, respectively (Figure 3). PERMANOVA on the broccoli samples alone further corroborated that both SFN-NIT (*p* = 0.0089) and IBN-NIT (*p* = 0.009) were significantly different between the two clusters, indicating that differing microbiome compositions found in each cluster yielded differing metabolic capabilities. These results indicate that the composition of the gut microbiome influences conversion of GLS to NITs.

### 3.4. Members of Family Clostridiaceae Are Associated with Nitrile Production

To examine which bacteria are associated with each microbial community (cluster) identified through our beta-diversity analysis, and thus NIT abundance, we conducted discriminant, differential abundance, and correlation analyses. Linear discriminant analysis effect size (LEfSe) was used to identify which bacterial taxa discriminate between high and low NIT producers, as represented in the two clusters. In the pre-incubation samples, the genera *Alistipes*, and families Rikenellaceae, and Ruminococcaceae were associated with low-NIT producers (cluster 2), while *Lachnoclustridium* was found to be associated with high-NIT production (cluster 1). In the post-incubation samples, LEfSe identified the family Enterobacteriaceae and the order Bacteriodales as being associated with the low-NIT producing (cluster 2) samples. Conversely, the phylum Firmicutes, specifically members of the Clostridia order and Clostridiaceae family, were associated with high-NIT producing individuals (cluster 1).

To verify these results in an independent manner and further identify individual taxa differentially abundant between high- and low-NIT producers, a negative binomial model was built. In the pre-incubation samples, 30 taxa were significantly different, however, only four taxa had a log_2_ fold-change value of greater than 1.5. The genera *Akkermansia, Alistipes,* and *Flavonifractor* were found to be more abundant in the low-NIT producers, while *Butyricoccus* was found to be more abundant in the high-NIT producers. In the post-incubation samples, seven taxa were significantly different (*p* < 0.05) between the observed clusters, all of which belonged to the families Enterobacteriaceae, Enterococcaceae, Clostridiaceae, and Coriobacteriaceae (Table 1). Members of Enterobacteriaceae were significantly more abundant in low-NIT individuals, while taxa from Clostridiaceae and Coriobacteriaceae were more abundant in high-NIT individuals. Members of family Enterococcaceae were found to be more abundant in low-NIT producers in NC samples, but more abundant in high-NIT producers. These findings align with what we observed in our LEfSe analysis and support the notion that the bacterial compositions differed between the two communities observed and may be associated with NIT levels.

To identify potential relationships between individual bacterial families and NIT abundance, Spearman’s correlations between the abundance of each family and the abundance of NIT across all Broc samples were performed (Figure S3). This model was naïve to clustering observed through beta-diversity analysis. In the pre-incubation samples, the Enterobacteriaceae family was once again found to have a strong negative correlation with SFN-NIT (rho = −0.541) and IBN-NIT (rho = −0.486). Interestingly, Clostridiaceae had very weak negative correlations with SFN-NIT (rho = −0.261) and IBN-NIT (rho = −0.03) and Enterococcaceae had a moderate negative correlation with SFN-NIT (rho = −0.558) and IBN-NIT (rho = 0.485). Post-incubation, Enterobacteriaceae was again found to be strongly negatively correlated with both SFN-NIT (rho = −0.758) and IBN-NIT (rho = −0.709), while the Clostridiaceae family was strongly positively correlated with SFN-NIT (rho = 0.673) and IBN-NIT (rho = −0.697). Enterococcaceae had a moderately strong positive correlation with both SFN-NIT (rho = 0.406) and IBN-NIT (rho = 0.515). Taken together, the results of all three analyses support the notion that the presence of certain bacterial taxa are associated with differential metabolite production, such as NITs.

### 3.5. SFN-NIT Variation Exists in Humans

**Figure 4:**
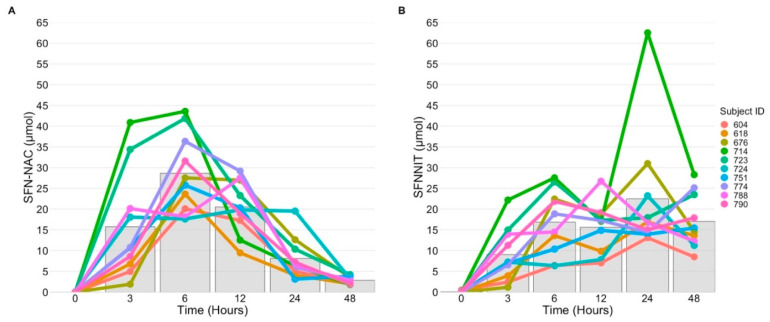
(**A**) Sulforaphane-N-acetyl cysteine (SFN-NAC) and (**B**) sulforaphane nitrile (SFN-NIT) detected in human urine following consumption of ~200 µmol SFN equivalents in fresh broccoli sprouts directly following the 0 h time point. Colored dots represent levels of SFN metabolites in each individual subject and solid bars represent mean mass of metabolites excreted (*n* = 10).

Data from our fecal culture experiments and results from others suggest that the microbiome may have an important role in how glucoraphanin is metabolized to SFN-NIT, but, to date, it is not clear how much SFN-NIT is excreted following broccoli sprout consumption by humans. To determine if the variations in SFN-NIT production we observed in fecal cultures are also observed with human broccoli consumption, we analyzed urine samples from participants fed a single dose of broccoli sprouts containing 200 µmol of SFN equivalents (full results published in [14]). Urine SFN-NIT and SFN-N-acetyl cysteine (SFN-NAC), the primary urinary metabolite of SFN, first appeared in the urine 3 h after consumption (Figure 4). SFN-NAC peaked at 6 h at 28.64 ± 2.99 µmol, and from 12 to 48 h SFN-NAC levels dropped continuously, reaching a mean of 2.84 ± 0.27 µmol at 48 h. SFN-NIT excretion showed greater variations between individuals. Six of 10 individuals had a peak in SFN-NIT excretion at 6 h following consumption, followed by a subsequent rise in SFN-NIT levels at 24 or 48 h following consumption. The other four individuals had a gradual increase in SFN-NIT excretion following consumption that peaked between 24 and 48 h. Mean excreted SFN-NIT levels were 16.86 ± 2.06 µmol at 6 h, 22.48 ± 4.77 µmol at 24 h, and 17.06 ± 2.07 µmol at 48 h.

## 4. Discussion

Human clinical trials utilizing broccoli sprouts, and other whole food interventions, frequently observe high levels of inter-individual variation between subjects, coinciding with equivocal efficacy [12,14,28,47,82,83,84]. The source of variation is still unknown, but a growing body of evidence points towards the gut microbiome as playing a key role in dictating the metabolic fate of GLS from whole foods. The role of the gut microbiome on GLS metabolism, using dynamic and complex systems, is still relatively undescribed and the human excretion of NITs, a GLS metabolite considered to be biologically inert, from broccoli sprouts has yet to be measured. Utilizing urine samples from humans fed 200 µmol of SFN-equivalents in broccoli sprouts, we detected high levels of inter-individual variation in SFN-NIT excretion following consumption. Using an ex vivo fecal incubation system, we determined that the gut microbiome influences production of NITs from broccoli sprouts. Specifically, we found that individuals with gut microbiomes enriched with members of the family Clostridiaceae had relatively high levels of SFN-NIT and IBN-NIT, while those with gut microbiomes abundant with taxa from the family Enterobacteriaceae had a relatively low abundance of NITs. Cumulatively, these findings point towards the gut microbiome as playing a key role in influencing the production of NITs from broccoli sprouts. Understanding the factors which drive variation in GLS metabolism may explain why some individuals benefit more greatly from the anti-cancer properties of cruciferous vegetables than others.

As expected, we found that anaerobic incubation significantly altered the composition of bacterial populations. Incubation with in vitro digested broccoli sprouts, as compared to the control digest, resulted in no significant alterations to the bacterial community. Previous research conducted in animal models and humans have shown that consumption of cruciferous vegetables can significantly alter the composition of the gut microbiome and prolonged consumption has been shown to “prime” the gut microbiome to more efficiently hydrolyze GLSs to ITCs, increasing bioavailability [46,47,52]. The lack of alteration observed in our study may be due to the relatively short exposure of our ex vivo microbiomes to broccoli as compared to other studies which occurred over weeks [46,85]. Alternatively, the first few hours of incubation may be most pivotal in establishing the microbial communities we observed and, thus, by only sampling at one later time point we missed the presence of important taxa. Future time-course experiments will be important to answering these outstanding questions.

In addition to high inter-individual variation in NIT production in humans, using an ex vivo fecal incubation model, we observed that the composition of the gut microbiome influenced the production of NITs from GLS. It is well known that in the absence of plant myrosinase, such as in cooked vegetables, members of the human commensal gut microbiota can hydrolyze glucosinolates to both bioactive ITCs and biologically inert NITs. Initial experiments on the microbial metabolism of GLS indicated that acidic conditions and the presence of Fe^2+^ atoms led to the formation of NITs as opposed ITCs [3,4,86,87,88,89]. Contrary to these findings, recent evidence has shown specific members of the gut microbiome produce NITs regardless of pH, suggesting the presence of an alternative metabolic pathway dependent on the composition of the gut microbiome [48,49,90,91]. We observed that post-incubation samples enriched with members of the Enterobacteriaceae and possibly Enterococcaceae families contained relatively low levels of NITs compared to those enriched in taxa from the Clostridiaceae family. These results align with in vitro studies using GLS isolates in monoculture systems to explore the metabolic capabilities of different microbes. In these studies, it was found that members of the Enterococcaceae and Enterobacteriaceae families were shown to be capable of degrading GLS to both ITCs and NITs [48,49,50,51]. One member, *Enterococcus casseliflavus*, a bacteria of the family Enterococcaceae, produced solely ITCs and another study found that cabbage inoculated with bacteria from the Enterococcaceae and Enterobacteriaceae families produced primarily ITCs, even under acidic conditions where NIT production is favored [49,51]. While our study found that samples that contained relatively low abundance of NITs were enriched with specific ASVs from Enterococcaceae, overall the Enterococcaceae family was positively correlated with the abundance of NITs. These contradictory findings highlight the complexity of microbial systems and how taxonomic relationships may not necessarily inform on metabolic capability, a major limitation of 16S sequencing and analysis. An important area of future investigations is the use of metagenomic sequencing methods to capture not only taxonomy, but also functional capacity. Alternatively, metabolomics can be coupled with 16S sequencing to capture metabolic-intermediates of GLS metabolism and identify the metabolic pathways enriched by broccoli sprout consumption.

We likewise found that individuals with a high abundance of NITs had microbiomes, in post-incubation samples, which were enriched with taxa from the family Clostridiaceae. One hypothesis of the metabolic pathways that microbes use to convert GLS to NIT is through the conversion of GLS to desulfoglucosinolates (DSGLS), which are then hydrolyzed to NITs [49,50,86,90,91]. The sulfatase gene required for conversion of GLS to DSLGS has yet to be identified, however, members of the Clostridiaceae family are known to contain sulfatases, which could potentially fill this metabolic niche [91,92]. These results are corroborated by a study, which found that consumption of a diet rich in glucosinolates resulted in a decrease in sulfate-reducing bacteria [54]. Sulfate is a product of GLS conversion to DSGLS, and thus may act as a metabolic substrate for sulfate-reducing bacteria. Unfortunately, the authors did not evaluate the production of GLS, DSGLS, or any ITC metabolites. A recently published study by Charron et al. found that individuals with BMIs greater than 26 kg/m^2^ absorbed significantly less SFN-metabolites than those with BMIs less than 26 kg/m^2^ [82]. While the authors did not examine the gut microbiomes of their participants, enrichment of bacteria from the Firmicutes phylum was associated with both a negative metabolic profile and obesity [82].

We repeatedly identified members of the Clostridiaceae family, belonging to the Firmicutes phylum, as being enriched in high-NIT producers, a finding which could explain the results found in the Charron et al. study. Another study by Kaczmarek et al. found that the consumption of broccoli led to a decrease in bacteria from the Firmicutes phylum, and that these bacteria had a negative correlation with the maximum peak of ITC-metabolites detected in plasma [53]. This is consistent with our results, although we did not measure ITCs; the production of NITs may shunt GLS away from conversion to ITCs. Unfortunately, general health information regarding the fecal donors from our study was not available and, thus, the impact of obesity on the gut microbiome cannot be accounted for in our study. An emphasis on the influence of biological factors, such as BMI, pharmaceutical use, and habitual diet, on gut microbiome composition and GLS metabolism to ITC and NITs will be an important area of future research. Interestingly, a study in mice fed broccoli found a positive correlation between Clostridiaceae and ITC abundance, further highlighting the importance of using metagenomic analyses to understand the role of the gut microbiome or integrating 16S sequencing with metabolomics data to further bridge the gap between taxonomy and function [93].

To the best of our knowledge, this is the first study to measure SFN-NIT levels in humans following consumption of broccoli sprouts. We found high levels of inter-individual variation in SFN-NIT excretion between subjects. One study examined the conversion of 150 µmol of purified glucoraphanin to SFN and SFN-NIT in rats by oral gavage [94]. The authors of that study found that SFN-NIT accounted for under 2% of the given dose of glucoraphanin [94]. By contrast, our study found that the total SFN-NIT excretion in urine accounted for approximately 40% of the given dose. The discrepancy between these findings could stem from the use of purified glucoraphanin in the absence of myrosinase used in the rat study, as opposed to the use of whole fresh broccoli sprouts fed with other food components to the humans in our study. In our study, we additionally found that the excretion of SFN-NIT varied greatly between individuals, with some individuals excreting a high amount at 6 h, as well as 24 and 48 h, while other subjects only experienced the highest level of excretion between 24 and 48 h. The most likely explanation for this finding is enterohepatic circulation of un-hydrolyzed glucoraphanin. The previously mentioned study by Bheemreddy and Jeffery found that glucoraphanin underwent enterohepatic circulation and was excreted into the gut through the bile duct [94]. In our study, we observed the highest levels of variation in NIT production at the 24 h timepoint, a finding which could potentially coincide with variable compositions of the gut microbiome. 

Cumulatively, these results indicated that the composition of the gut microbiome could play a key role in dictating the bioavailability and generation of bioactive ITCs from broccoli sprouts. High production of NITs could shunt GLS away from being converted to ITCs, thus leading to decreased efficacy due to the lower absorption of the bioactive anti-cancer compounds from broccoli sprouts. Further work conducted on humans is required to understand the role of the gut microbiome in both ITC and NIT generation.

## Figures and Tables

**Table 1:** ASVs differentially abundant between high and low nitrile producers.

ASV	Term *	Adjusted *p*-Value	Log2 Fold Change ^§^	Phylum	Class	Order	Family	Genus
ASV1	NC	1.95 × 10^−207^	−8.03	Proteobacteria	Gammaproteobacteria	Enterobacteriales	Enterobacteriaceae	*Escherichia/Shigella*
ASV1	Broc	1.13 × 10^−281^	−10.85	Proteobacteria	Gammaproteobacteria	Enterobacteriales	Enterobacteriaceae	*Escherichia/Shigella*
ASV49	NC	2.47 × 10^−21^	−1.51	Firmicutes	Bacilli	Lactobacillales	Enterococcaceae	*Melissococcus*
ASV49	Broc	3.60 × 10^−03^	2.02	Firmicutes	Bacilli	Lactobacillales	Enterococcaceae	*Melissococcus*
ASV556	Broc	7.54 × 10^−46^	3.05	Firmicutes	Clostridia	Clostridiales	Clostridiaceae_1	*f_Clostridiaceae_1_ASV556*
ASV683	Broc	5.77 × 10^−38^	2.95	Firmicutes	Clostridia	Clostridiales	Clostridiaceae_1	*f_Clostridiaceae_1_ASV683*
ASV872	Broc	8.84 × 10^−28^	4.26	Actinobacteria	Coriobacteriia	Coriobacteriales	Coriobacteriaceae	*Collinsella*

* Treatment group significant difference found. ^§^ High nitrile producers vs low nitrile producers.

## Data Availability

Datasets of the current study are not publicly available, but available upon request.

## Supplementary Materials

The following are available online at www.mdpi.com/article/10.3390/nu13093013/s1, Figure S1: Rarefaction curves of 16S Sequenced Samples, Figure S2: PCoA of only Broc and NC samples, Figure S3: Spearman’s Correlations between family abundance and SFN-NIT and IBN-NIT levels, Table S1: Alpha-diversity measures for all samples, Table S2: Differences in alpha-diversity by different metadata factors, Table S3: Measures of alpha-diversity of each treatment group pre- and post-incubation.
