# Supplementary figures and images for: Composition of the Gut Microbiome Influences Production of Sulforaphane-Nitrile and Iberin-Nitrile from Glucosinolates in Broccoli Sprouts
Source: Nutrients. 2021 Aug 28;13(9):3013. doi: 10.3390/nu13093013 (PMC8468500; doi:10.3390/nu13093013)

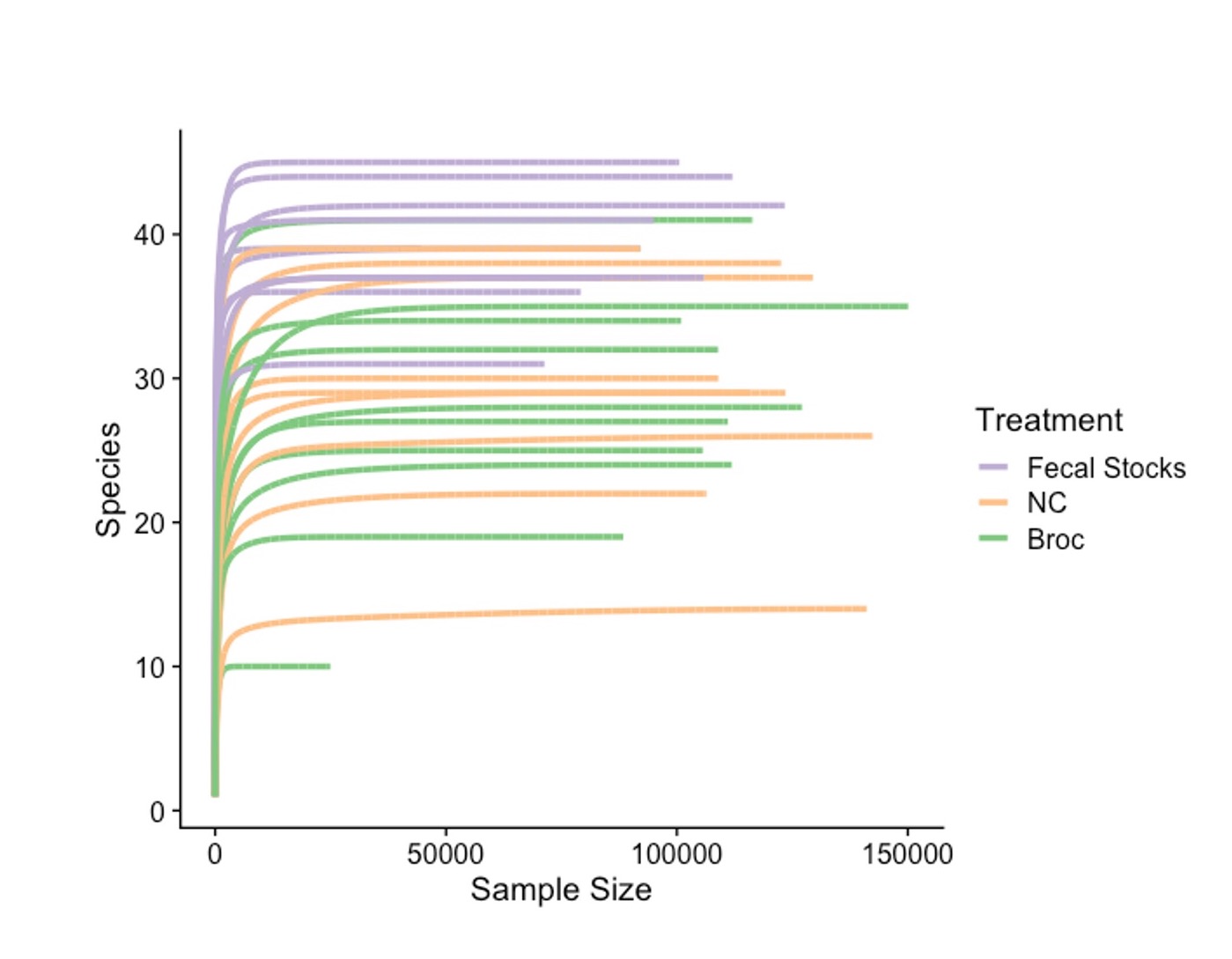

Supplement: Supplementary file 1 [file nutrients-13-03013-s001.zip › Nutrients_Supplemental/Figure_S1.jpg]

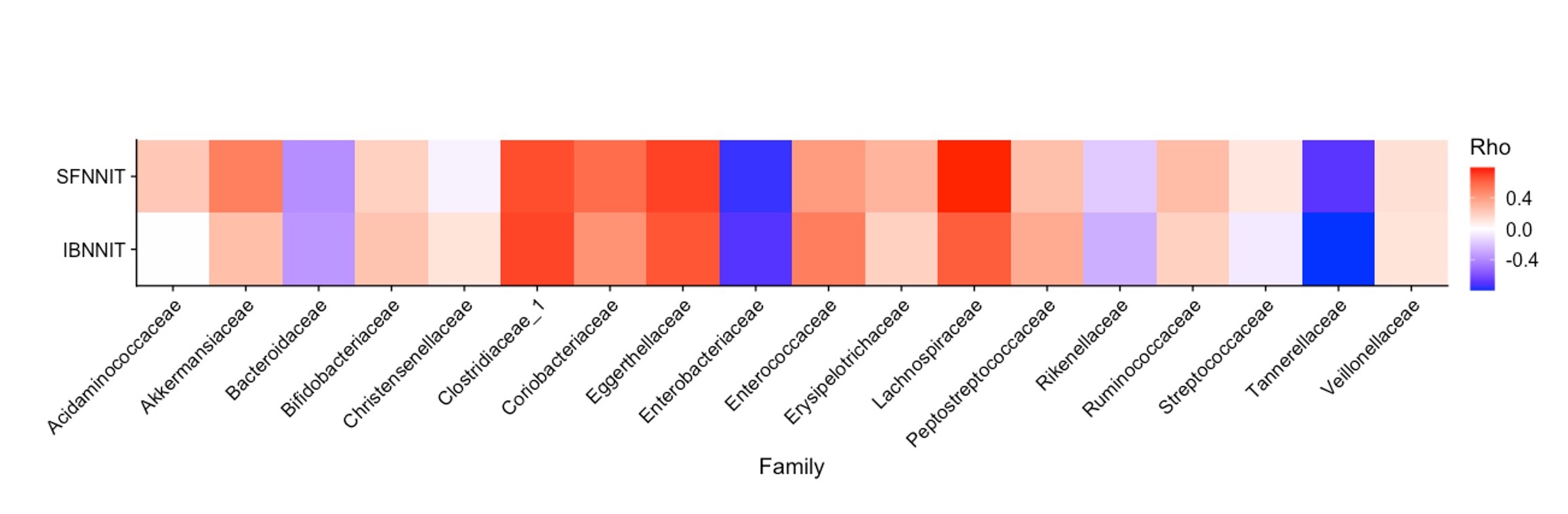

Supplement: Supplementary file 1 [file nutrients-13-03013-s001.zip › Nutrients_Supplemental/Figure_S3.jpg]

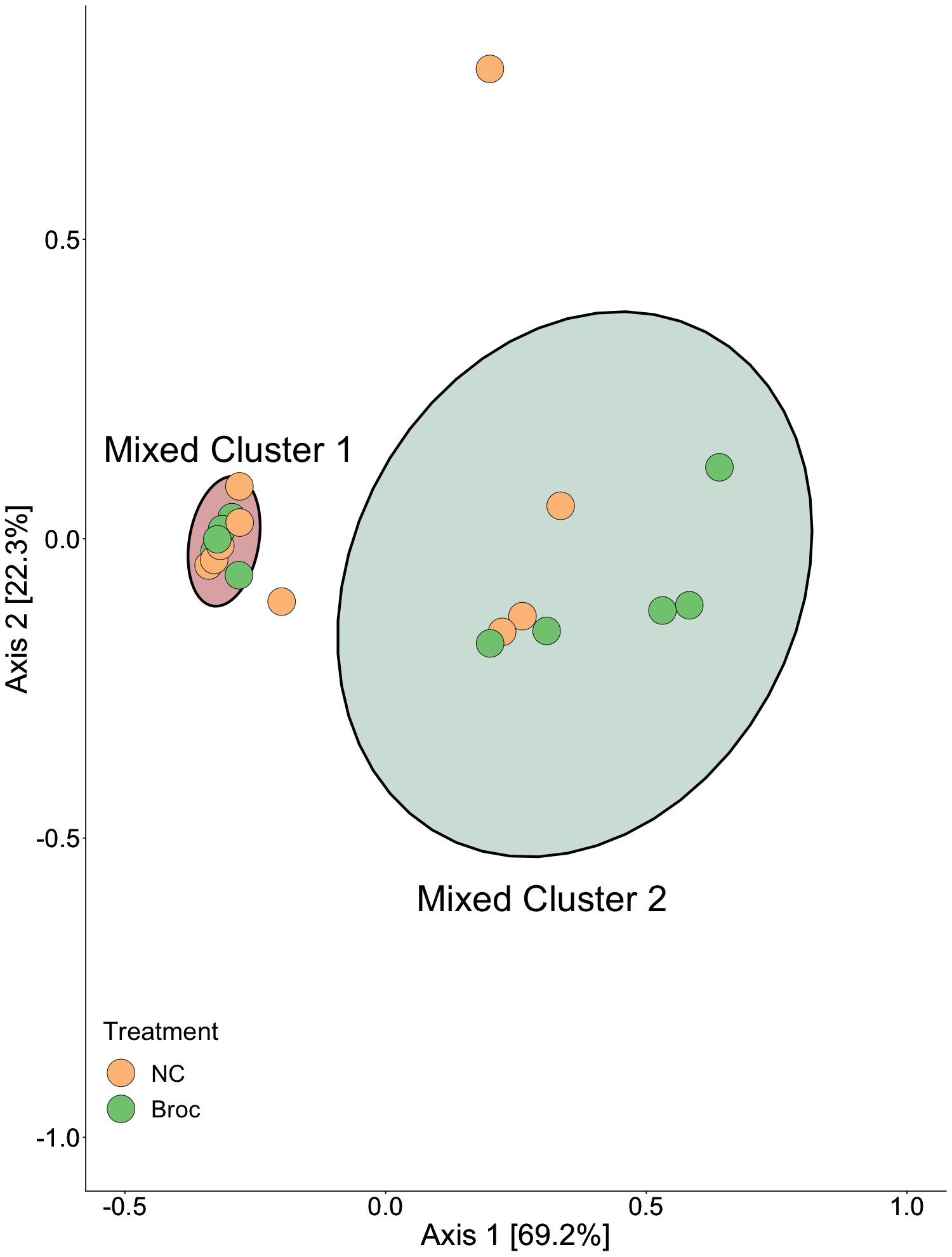

Supplement: Supplementary file 1 [file nutrients-13-03013-s001.zip › Nutrients_Supplemental/Figure_S2.jpeg]
